# Design aspects of COVID‐19 treatment trials: Improving probability and time of favorable events

**DOI:** 10.1002/bimj.202000359

**Published:** 2021-10-22

**Authors:** Jan Beyersmann, Tim Friede, Claudia Schmoor

**Affiliations:** ^1^ Institut für Statistik Universität Ulm Ulm Germany; ^2^ Institut für Medizinische Statistik Universitätsmedizin Göttingen Göttingen Germany; ^3^ Deutsches Zentrum für Herz‐Kreislaufforschung (DZHK) Standort Göttingen Göttingen Germany; ^4^ Zentrum Klinische Studien, Universitätsklinikum Freiburg, Medizinische Fakultät Albert‐Ludwigs Universität Freiburg Freiburg im Breisgau Germany

**Keywords:** clinical trials, competing events, COVID‐19, outcomes, SARS‐CoV‐2

## Abstract

As a reaction to the pandemic of the severe acute respiratory syndrome coronavirus 2 (SARS‐CoV‐2), a multitude of clinical trials for the treatment of SARS‐CoV‐2 or the resulting corona disease 2019 (COVID‐19) are globally at various stages from planning to completion. Although some attempts were made to standardize study designs, this was hindered by the ferocity of the pandemic and the need to set up clinical trials quickly. We take the view that a successful treatment of COVID‐19 patients (i) increases the probability of a recovery or improvement within a certain time interval, say 28 days; (ii) aims to expedite favorable events within this time frame; and (iii) does not increase mortality over this time period. On this background, we discuss the choice of endpoint and its analysis. Furthermore, we consider consequences of this choice for other design aspects including sample size and power and provide some guidance on the application of adaptive designs in this particular context.

## 1 INTRODUCTION

At the time of writing, the severe acute respiratory syndrome coronavirus 2 (SARS‐CoV‐2) pandemic is ongoing. As a reaction to the pandemic, a multitude of clinical trials on the treatment of SARS‐CoV‐2 or the resulting corona disease 2019 (COVID‐19) are globally in planning, were recently initiated or already completed. Although some attempts were made to standardize study designs, this was hindered by the ferocity of the pandemic and the need to set up clinical trials quickly.

For randomized controlled trials evaluating the safety and efficacy of COVID‐19 treatments, the choice of appropriate outcomes has received considerable attention in the meanwhile. For instance, WHO (2020) suggests an eight‐point ordinal scale as part of their master protocol while Dodd et al. (2020) discuss the use of survival methodology to investigate *both* an increase of the event probability of a favourable outcome such as improvement or recovery *and* its timing. Similar to McCaw et al. (2020b), Dodd et al. stress that such outcomes are subject to competing risks or competing events, because patients may die without having achieved the favourable outcome. The authors argue that these patients must not be censored at their time of death but, say, at day 28 after treatment, if the trial investigates a 28‐day‐follow‐up. The authors continue to advocate investigating hazard ratios based on such data (called improvement or recovery rate ratio by the authors, because the outcome is not hazardous to health, but favourable). We will use improvement and recovery interchangeably from a methodological perspective as examples for favorable events (although clinically they are of course different). Furthermore, Benkeser et al. (2020), an early methodological publication on COVID‐19, consider time‐to‐event outcomes in a paper advocating covariate adjustment in randomized trials, but do not consider competing events. Rather, the authors consider a composite of intubation and death, thereby avoiding the need to model competing events. This composite combines two unfavourable outcomes, but for outcomes recovery or improvement, such a composite endpoint that also includes death is not meaningful.

One aim of our paper is to provide an in‐depth treatment of why one typically needs to account for competing events in a time‐to‐improvement or time‐to‐recovery analysis of COVID‐19 treatment trials. Here, we will start with a clear definition of the target parameters rather than with censoring rules and connect them with the aims of a successful treatment of COVID‐19 patients. To begin, the role of censoring here is subtle: Dodd et al. (2020) state that the improvement or recovery rate ratio approach coincides with the subdistribution hazard ratio approach of Fine and Gray (1999), *if* there is additional, “usual” censoring as a consequence of staggered study entry. McCaw et al. (2020b), on the other hand, characterize the approach to censor previous deaths at day 28, still assuming a 28‐day‐follow‐up, as “unusual” and warn against the use of one minus Kaplan–Meier, *if* there is additional censoring before day 28, say, because of staggered entry. In our presentation of motivating examples in Section 2, we will see that both Kaplan–Meier estimates censoring at the time of death and Kaplan–Meier estimates censoring at day 28 are being used in COVID‐19 trials.

Recently, Kahan et al. (2020) discussed outcomes in the light of the estimand framework. Another important aim of our paper is to clarify and provide guidance with respect to the target parameters when using survival methodology to investigate *both* an increase of the event probability of a favourable outcome *and* its timing. To this end, we will demonstrate that censoring deaths on day 28 in a trial with a 28‐day‐follow‐up conceptually corresponds to formalizing time to improvement or recovery via improper failure times, which we will call subdistribution times, with probability mass at infinity. The latter corresponds to the probability of death during 28‐day or, more generally, τ‐day‐follow‐up. This has various consequences: It allows to formalize mean and median times to improvement (recovery). The Kaplan–Meier estimator based on death‐censored‐on‐day‐τ‐data will coincide with the Aalen–Johansen estimator of the cumulative event probability considering the competing event death, provided that there is no additional censoring. The hazard ratio at hand will be a subdistribution hazard ratio as a consequence of using subdistribution times, but not as a consequence of additional censoring.

In this article, we take the view that a successful treatment of COVID‐19 patients (i) increases the probability of a recovery or improvement within a certain time interval, say 28 days; (ii) aims to expedite recovery or improvement within this time frame; and (iii) does not increase mortality over this time period (see, e.g., Wilt et al., 2020). This should be reflected in the main outcomes of a COVID‐19 treatment trial. The choice of outcomes has also some implications for the trial design. First, even in traditional designs the sample size calculation might be complicated by the presence of competing events. Second, novel trial designs including platform trials and adaptive group‐sequential designs are more frequently applied than usual in COVID‐19 treatment trials. Stallard et al. (2020) provide an overview over such designs, discuss their utility in COVID‐19 trials, and make some recommendations. In the light of the outcome discussion, we provide some comments on the application of such outcomes in adaptive designs. While our paper has a clear focus on COVID‐19 treatment trials, the considerations apply more generally if time‐to‐event is of interest, where “time” is measured within a rather restricted fashion such as 28 days, but “event” is subject to competing risks. As a rule of thumb, for instance, hospital outcomes in severely ill patients will generally fit this pattern.

The article is organized as follows. In Section 2, background on some example trials is provided to motivate the investigations presented here. In Section 3, outcomes, their analysis, and interpretation are considered before some guidance is provided on planning such trials in Section 4. We close with a discussion in Section 5.

## 2 MOTIVATING EXAMPLES

Our starting point is that a successful treatment of COVID‐19 patients (i) increases the proportion of recoveries within a time interval [0,τ], say, τ=28 days; (ii) aims to expedite recovery on [0,τ]; and (iii) does not increase mortality at time τ. Aim (i) is obviously desirable both from a patient's perspective and from a public health perspective. The rationale behind aim (ii) is that two different treatments that lead to comparable recovery proportions at time τ may differ in the timing of recoveries. Here, faster recovery is not only desirable from a public health perspective with respect to available resources, but faster recovery from ventilation will also benefit the individual patient. Finally, the requirement (iii) reflects that a treatment that increases both the proportion of recoveries and the proportion of deaths at time τ benefits some patients and harms others.

We will argue that aims (i)–(iii) cast COVID‐19 trials into a competing events (or competing risks) setting, although this is not necessarily or not explicitly recognized. For example, the primary clinical endpoint of Wang et al. (2020) was time for clinical improvement within 28 days after randomization, addressing aims (i) and (ii). Within τ=28 days, 13% of the patients in the placebo group and 14% in the treatment (Remdisivir) group died. The authors aimed to address such competing mortality before clinical improvement by right‐censoring time to clinical improvement at τ for patients dying before τ. The authors then used the usual machinery of Kaplan–Meier, log‐rank, and Cox proportional hazards regression. However, as we will see below, their analysis amounts to using the Aalen–Johansen estimator of the cumulative event probability (instead of Kaplan–Meier), Gray's test for comparing cumulative event probabilities (or subdistributions) between groups (instead of the common log‐rank test) and Fine and Gray's proportional subdistribution hazards model (instead of the usual Cox model) (Beyersmann et al., 2012).

Other recent examples are Beigel et al. (2020) who consider time to recovery on days [0,28] and Cao et al. (2020) whose primary outcome is time to clinical improvement until day 28. Beigel et al. also censor previous deaths “on the last observation day” (see the Appendix of Beigel et al.) and use Kaplan–Meier (here, actually, Aalen–Johansen) and log‐rank test (here, actually, Gray's test) to analyze these data. Cao et al. censor both “failure to reach clinical improvement or death before day 28” on day 28 and use Kaplan–Meier, log‐rank, and the Cox model (here, actually, Fine and Gray). Interestingly, Cao et al. comment that “right‐censoring occurs when an event may have occurred after the last time a person was under observation, but the specific timing of the event is unknown,” although this is clearly not the case for patients censored at day 28 following the death before that time.

To be precise, let ϑ be the time to clinical improvement, using the example of Wang et al. Time to improvement is, in general, *not* well defined for patients dying prior to improvement. To address this, the subdistribution time is defined as ϑ=∞ for the latter patients, that is,

$$\vartheta \;\left\{\begin{array}{cc} <\infty & \text{the}\;\text{outcome}\;\text{is}\;\text{reached,}\\ =\infty & \text{death}\;\text{occurs}\;\text{before}\;\text{the}\;\text{outcome}\;\text{is}\;\text{reached.} \end{array}\right.$$

The interpretation of the improper random variable ϑ is that it equals the actual time of improvement when ϑ<∞. However, patients who die before improvement will never experience this primary outcome and, hence, ϑ=∞. The censored subdistribution time in the paper of Wang et al. becomes

$$\tilde{\vartheta }=\min\left(\vartheta ,\tau \right).$$

It is instructive to reexpress matters in standard competing risks notation with time‐to‐first‐event T and type‐of‐first‐event ε. Recall that we are assuming that censored patients are either censored because of death before day τ or because they have neither reached the outcome event nor have died on [0,τ]. This situation is a special case of “censoring complete” data (Fine & Gray, 1999), which means that the potential censoring time is known for all patients.

We will investigate the consequences of competing events in the following sections, including alternatives to the subdistribution framework, the need to still analyze competing mortality, and possible strategies to account for death *after* recovery. Here, we stress that the aim to account for our items (i)–(iii) above has led authors to implicitly employ a competing event analysis, although this is not explicitly acknowledged. One worry is that the subdistribution hazards framework has repeatedly been reexamined, questioning the interpretability of a hazard belonging to an improper random variable (Andersen & Keiding, 2012). The key issue here is that patients are still kept “at risk” after death and until censoring at τ, although, of course, no further events will be observed for these patients.

There are further examples of the presence of competing events in COVID‐19 studies. For instance, Grein et al. (2020) use Kaplan–Meier for time‐to‐clinical improvement at day 28. These authors report a Kaplan–Meier estimate of 84% for the cumulative improvement probability at τ=28, although the improvement was only observed for 36 (68%) out of 53 patients. Letters to the Editor and a Reply by Grein et al. reveal that the original Kaplan–Meier analysis had censored deaths before improvement at the time of death, but not at τ. It is well known that such an analysis is subject to “competing risks bias” and must inevitably overestimate cumulative event probabilities (Beyersmann et al., 2012).

As the last example of this section, we consider ventilator‐free days (VFDs) (see Schoenfeld et al., 2002 and Yehya et al., 2019), which is the primary or secondary outcome in a number of ongoing trials (trial identifiers NCT04360876, NCT04315948, NCT04348656, NCT04357730, NCT03042143, NCT04372628, NCT04389580 at clinicaltrials.gov, and DRKS00021238 at clinicaltrialsregister.eu). Yehya et al. provide an applied tutorial on using VFDs as an outcome measure in respiratory medicine. Similar to our aims (i)–(iii) above, they argue in favor of using VFDs, because they “penalize nonsurvivors,” that using time as an outcome “provide[s] greater statistical power to detect a treatment effect than the binary outcome measure” and that time is relevant in that “shortened ventilator duration is clinically and economically meaningful.” Again, censored subdistribution times are present, although this is not made explicit in the definition of VFDs. Choosing once more a time horizon of τ=28 days, VFDs are defined as 28−x if ventilation stops on day x, but are defined as 0 if the patient either dies while being ventilated or is still alive and ventilated after [0,28]. Interpreting the subdistribution time ϑ as the day when ventilation is stopped while alive (and not as a consequence of death), we get

$$\text{VFDs}=28-\tilde{\vartheta }.$$

Consequently, Yehya et al. also suggest the proportional subdistribution hazards model as one possible statistical analysis.

## 3 OUTCOMES, THEIR ANALYSIS AND INTERPRETATION

**FIGURE 1:**
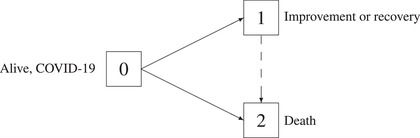
Competing events model (solid arrows only) and illness–death model without recovery (solid and dashed arrows) for outcomes improvement or recovery in the presence of the competing event death

Consider a stochastic process ${\left(X\left(t\right)\right)}_{t\in [0,\tau ]}$ with state space {0,1,2}, right‐continuous sample paths and initial state 0, P(X(0)=0)=1 (see Figure 1). Patients alive with COVID‐19, randomized to treatment arms, are in state 0 of the figure at time 0.

Our main model will be the competing events model in Section 3.1, considering only the solid arrows in Figure 1. Improvement or recovery is modeled by a 0→1 transition, death without prior improvement (or recovery) is modeled by a 0→2 transition. In practice, for example, improvement is typically defined as improving by one or two categories on the eight‐point ordinal scale for clinical improvement proposed in the master protocol of the WHO (2020) ; hence, a 0→1 transition occurs at the time of such improvement.

Later, in Section 3.4, we will briefly consider an extension of this model to an illness–death model without recovery by also considering 1→2 transitions, that is, death after improvement events. This is illustrated in Figure 1 by the dashed arrow. Note that “illness–death without recovery” does not mean that recovery may not be modeled, but that 1→0 transitions are not considered. In terms of outcomes, X(t)=1 in the competing events model means that improvement has occurred on [0,t], but in the illness–death model, X(t)=1 means that improvement has occurred *and* that the patient is still alive. The distinction may be relevant for trials in patients where possible subsequent death on [0,τ] is a concern; see Sommer et al. (2018) for a discussion of death after clinical cure in treatment trials for severe infectious diseases. An extension of such a multistate model would also allow to model transitions between categories such as those of the WHO blueprint scale for clinical improvement.

Returning to the goals of a successful treatment of COVID‐19 patients mentioned earlier, goal (i) is reflected by an increased state occupation probability for state 1 in Figure 1, that is, an increased probability of recovery or improvement. Goals (ii) and (iii), earlier recovery and no increased mortality, are reflected by faster 0→1 transitions and no increased state occupation probability for state 2. Below, goal (ii) to expedite favorable events will be reflected using hazards.

### 3.1 Competing events: Time to and type of first event

Both in the competing events and, later, in the illness–death model, time‐to‐first‐event is

(1) T=inf{t:X(t)≠0},

the waiting time in state 0 of Figure 1, with type‐of‐first‐event ε=X(T),

(2) X(T)∈{1,2},

the state the process enters upon leaving the initial state. The tupel (T,X(T)) defines a *competing events* situation. Note that competing events are characterized by time‐to‐first‐event and type‐of‐first‐event; it is not assumed that there are no further events after a first event. However, the analysis of subsequent events requires more complex models such as an illness–death model.

The stochastic process for time and type of the first event is regulated by the event‐ (or cause‐) specific hazards

(3) $$\alpha_{0j}\left(t\right)=\lim_{\Delta t↘0}\frac{P(T\in [t,t+\Delta t),X(T)=j\;|\;T\geq t)}{\Delta t},\;j\in \left\{1,2\right\},t\in \left[0,\tau \right],$$

which we assume to exist. Their sum

$$\alpha_{0\cdot }\left(t\right)=\alpha_{01}\left(t\right)+\alpha_{02}\left(t\right)$$

is the usual all‐events hazard of time T with survival function

$$P\left(T>t\right)=\exp\left(-\int_{0}^{t}\alpha_{0\cdot }\left(u\right)\;du\right).$$

Note that T is the time until a composite of improvement or death, whatever comes first, which is *not* a meaningful outcome in the present setting, combining an endpoint that benefits the patient with one that harms the patient. Rather, as discussed above, authors consider the cumulative improvement probability

(4) $$F_{1}\left(t\right)=P\left(T\leq t,X\left(T\right)=1\right)=\int_{0}^{t}P\left(T\geq u\right)\alpha_{01}\left(u\right)\;du$$

(5) =1−P(ϑ>t),

with subdistribution time ϑ until improvement defined as

$$\vartheta =\left\{\begin{array}{cc} T & X(T)=1\\ \infty & X(T)=2. \end{array}\right.$$

Later, Equations (4) and (5) will resurface in that we will find that one minus Kaplan–Meier estimation of 5 will coincide with the standard Aalen–Johansen estimator of 4 for “censoring complete” data as described earlier.

A binary outcome, say improvement status at time τ, is covered by this framework,

1(T≤τ,X(T)=1)∈{0,1},

with indicator function 1(·) and improvement probability (at time τ) P(T≤τ,X(T)=1). However, viewing quantities (4) as a function of time allows to detect earlier improvement with possibly comparable improvement probabilities at τ. The expected proportion of deaths at τ without prior improvement is P(T≤τ,X(T)=2).

Key quantities of the competing events model are time and type of first event, (T,X(T)) and the event‐specific hazards $\left(\alpha_{01}\left(t\right),\alpha_{02}\left(t\right)\right)$. The subdistribution time ϑ appears to be little more than an afterthought of (4). However, its relevance is closely connected to the subdistribution *hazard* which equals neither any of the event‐specific hazards nor the all‐events hazard. It also reappears in the context of “average” improvement or recovery times, see Section 3.3.

The subdistribution hazard λ(t) is the hazard “attached” to (4) by requiring

(6) $$P\left(T\leq t,X\left(T\right)=1\right)=1-\exp\left(-\int_{0}^{t}\lambda \left(u\right)\;du\right),$$

leading (Beyersmann et al., 2012) to

(7) $$\lambda \left(t\right)={\left(1+\frac{P(T\leq t,X(T)=2)}{P(T>t)}\right)}^{-1}\alpha_{01}\left(t\right),$$

which illustrates why interpretation of the subdistribution hazard as a hazard has been subject of debate (Andersen & Keiding, 2012). The event‐specific hazards $\alpha_{0j}\left(t\right)$ have the interpretation of an instantaneous “risk” of a type j event at time t given one still is in state 0 just prior to time t. They may be visualized (Beyersmann et al., 2012) as forces moving along the solid arrows in Figure 1. There is no such interpretation for the subdistribution hazard. The above display also illustrates that a competing subdistribution hazard, “attached” to P(T≤t,X(T)=2), may not be chosen or modeled freely. This is in contrast to the event‐specific hazards.

The rationale of the subdistribution hazard is that it re‐establishes a one‐to‐one correspondence between (subdistribution) hazard and cumulative event probability (4), which otherwise is a function of both event‐specific hazards $\alpha_{01}\left(t\right)$ and $\alpha_{02}\left(t\right)$. The subdistribution hazard approach may also be viewed via a transformation model for (4) using the link x↦log(−log(1−x)) as in a Cox model for the all‐events hazard, but still without a common hazard interpretation. Several authors have argued in favor of link functions which are more amenable to interpretation such as a logistic link. (See also Section 4.1 on study planning with respect to odds ratios.) Against the background of the motivating examples in Section 2, we will put a certain emphasis on the former link but also note that it is not uncommon that results from either link function coincide from a practical point of view (Beyersmann & Scheike, 2014). In the absence of competing events, this has been well documented for studies with short follow‐up and low cumulative event probability (Annesi et al., 1989). In the present setting, trials will aim at increasing cumulative improvement or recovery probabilities but “competing” mortality implies that these probabilities must be below one, which distinguishes competing events from the all‐events framework.

### 3.2 Statistical approaches

We will assume that follow‐up data are complete in that improvement status and vital status is known for all patients on [0,τ]. In most of the motivating examples of Section 2, both patients alive but without improvement up to day τ and patients who had died were censored at τ. Although censored at this maximal time point, both improvement status and vital status are known for these patients on [0,τ]. Survival methodology discussed below will allow for right censoring of patients alive where further follow‐up information ceases at the time of censoring, but we assume that this is a minor problem when, for example, τ=28 days.

Assuming data to be complete in this sense, the Kaplan–Meier estimator of P(T>t) is

$$\hat{P}\left(T>t\right)=\prod_{u\leq t}\left(1-\frac{\Delta N(u)}{Y(u)}\right)=1-\frac{N(t)}{n},$$

where the product in the above display is over all unique event times, N(t) is the “all‐events” number of composite events (transitions out of the initial state in Figure 1) on (0,t], ΔN(t) is the number of events at time t, Y(t) is the number at risk just prior time t and n is the sample size. Because of complete follow‐up on [0,τ], $\hat{P}\left(T>t\right)$ equals the empirical event‐free fraction (n−N(t))/n.

Introducing

$$\begin{array}{ccc} N_{01}\left(t\right) & = & \text{Number}\;\text{of}\;0\to 1\;\text{transitions}\;\text{on}\;\left(0,t\right],\\ N_{02}\left(t\right) & = & \text{Number}\;\text{of}\;0\to 2\;\text{transitions}\;\text{on}\;\left(0,t\right],\\ N(t) & = & N_{01}\left(t\right)+N_{02}\left(t\right), \end{array}$$

with $\Delta N_{0j}\left(t\right)$ type j events precisely at time t, the Aalen–Johansen estimators are

$$\hat{P}\left(T\leq t,X\left(T\right)=j\right)=\sum_{u\leq t}\hat{P}\left(T\geq u\right)\frac{\Delta N_{0j}\left(t\right)}{Y(t)}=\frac{N_{0j}\left(t\right)}{n},\;j\in \left\{1,2\right\},$$

which also equal the usual empirical proportions assuming complete follow‐up. Here, one easily sees that

$$\hat{P}\left(T>t\right)+\hat{P}\left(T\leq t,X\left(T\right)=1\right)+\hat{P}\left(T\leq t,X\left(T\right)=2\right)=1,$$

a natural balance equation which is maintained even in the presence of censoring, but violated if one were to use

(8) $$1-\prod_{u\leq t}\left(1-\frac{\Delta N_{01}\left(u\right)}{Y(u)}\right)$$

to estimate the cumulative probability of a type 1 event (see the motivating examples of Section 2). This Kaplan–Meier‐type estimator inevitably overestimates, the reason being that one minus Kaplan–Meier approximates an empirical distribution function, but the cumulative probability of a type 1 event is bounded from above by P(X(T)=1). To this end, note that our notation always entails that $\hat{P}\left(T>t\right)$ is the Kaplan–Meier estimator of the proper event time T, T<∞, and $\hat{P}\left(T\leq t,X\left(T\right)=j\right)$ always is its generalization to the Aalen–Johansen estimator of the cumulative type j probability.

However, the Kaplan–Meier estimator predominantly used in the motivating examples of Section 2 is based on subdistribution times with censoring time τ leading to

$$\begin{array}{ccc} {\tilde{N}}_{01}\left(t\right) & = & \sum_{i}^{n}1\left(\vartheta_{i}\leq t\right),\\ \tilde{Y}\left(t\right) & = & Y\left(t\right)+\sum_{i}^{n}1\left(T_{i}<t,X\left(T_{i}\right)=2\right),t\leq \tau , \end{array}$$

where index i signals patient i, i∈{1,…n}. Because

$$1\left(\vartheta_{i}\leq t\right)=1\left(T_{i}\leq t,X\left(T_{i}\right)=1\right),$$

we have ${\tilde{N}}_{01}\left(t\right)=N_{01}\left(t\right)$, but $\tilde{Y}\left(t\right)\geq Y\left(t\right)$, because censoring dead patients at τ enlarges the risk set by the number of previous deaths. In the current setting, it is easy to demonstrate that the standard Aalen–Johansen estimator of the cumulative improvement probability accounting for the competing risk death equals one minus the Kaplan–Meier estimator based on the censored subdistribution times, that is,

(9) $$\hat{P}\left(T\leq t,X\left(T\right)=j\right)=1-\prod_{u\leq t}\left(1-\frac{\Delta N_{01}\left(u\right)}{\tilde{Y}\left(u\right)}\right),$$

(see the Appendix). Note that the difference between the right‐hand side of (9) and the biased Kaplan–Meier‐type estimator (8) lies in the use of a different risk set.

Any regression model for hazards may be fit to the event‐specific hazards, the most common choice being Cox models,

$$\alpha_{0j}\left(t;Z\right)=\alpha_{0j;0}\left(t\right)\cdot \exp\left(\beta_{0j}^{⊤}Z\right),\;j\in \left\{1,2\right\},$$

with event‐specific baseline hazards $\alpha_{0j;0}\left(t\right)$, event‐specific p×1 vectors of regression coefficients $\beta_{0j}$, and a p×1 vector of baseline covariates Z. Technically, an event‐specific Cox model for the type 1 hazard, say, may be fit by only counting type 1 events as events and by additionally censoring type 2 events at the time of the type 2 event. Roles reverse fitting an event‐specific Cox model for the type 2 hazard. For the interpretation, this has arguably been a source of confusion, since the biased Kaplan–Meier‐type estimator (8) also only counts type 1 events as events and additionally censors type 2 events. The difference between fitting an event‐specific Cox model and the Kaplan–Meier‐type estimator (8) is that *hazard* models allow for quite general censoring processes including censoring by a competing event. However, *probabilities* depend on all event‐specific hazards, which is why we have formulated Cox models for all event types above. It is, however, not uncommon to only see results from one event‐specific Cox model being reported; see Goldman et al. (2020) and Spinner et al. (2020) for two recent examples from COVID‐19 treatment trials.

In contrast to, for example, these two studies, event‐specific Cox models have not been used in the motivating examples above. Rather a Cox‐type model, the Fine and Gray model, for the subdistribution hazard has been employed,

$$\lambda \left(t;Z\right)=\lambda_{0}\left(t\right)\cdot \exp\left(\gamma^{⊤}Z\right).$$

If the cumulative improvement probabilities follow the Fine and Gray model, a subdistribution hazard ratio larger than one for treatment signals both an increase of the expected improvement proportion at τ and earlier improvement.

It has been repeatedly argued that any competing events analysis should consider all competing events at hand. For the event‐specific hazards, we have therefore formulated two Cox models. For the Fine and Gray approach, postulating a Cox‐type model for the “competing” subdistribution hazard is complicated by (7). However, delayed death on [0,τ] does not benefit the patient if τ=28 days. Hence, in the present setting, it will suffice to consider the probability P(X(T)=2) of “competing” probability by common methods for proportions.

### 3.3 “Average” improvement or recovery times

The subdistribution time ϑ is also useful for formalizing “average” improvement or recovery times. Assuming “competing” mortality, that is, P(T≤τ,X(T)=2)>0, it is easy to see that

E(ϑ)=∞,

because P(ϑ=∞)=P(X(T)=2). Consequently, the expected or mean time to improvement (recovery) is not a useful parameter. It is well known that in standard survival analysis (time‐to‐all‐causes‐death), expected survival time is typically not investigated, but for a different reason. For the latter, expected survival time is a finite number, but it is usually not identifiable, at least not in a nonparametric way, because of limited follow‐up. Both in this and in the present context, two possible solutions for investigating “average” times‐to‐event are restricted means and median times. For the former, Andersen (2013) considers

(10) $$\;E\left(\min(\vartheta ,\tau )\right)=\tau -\int_{0}^{\tau }F_{1}\left(u\right)\;du.$$

If the competing events are different causes of death, Andersen interprets τ minus (10) as the mean time span lost before time τ and “due to cause 1.” The area under the cumulative event probability

$$\left[0,\tau \right]∋t\mapsto F_{1}\left(t\right)$$

may hence be interpreted as the mean time from improvement (recovery) to time τ. For COVID‐19 trials, this parameter has recently been suggested by McCaw et al. (2020a), however, without giving formulae or making the link to subdistribution times explicit. This is also related to the ventilator free days discussed in Section 2. A recent example of using (10) is Hao et al. (2020) who considered influenza‐attributable life years lost before the age of 90.

Alternatively, one may consider the median time to improvement,

(11) $$\inf\left\{t\;:\;F_{1}\left(t\right)\geq 0.5\right\}.$$

Again, there is a conceptual difference to median survival time in that the latter always is a finite number (denying the possibility of immortality), but quantity (11) will be defined as infinity, if the eventual improvement probability does not reach 50%. However, if its Aalen–Johansen estimator does reach 50% on [0,τ], the median time‐to‐improvement can be estimated in a nonparametric fashion by plugging the Aalen–Johansen estimator into (11), see Beyersmann and Schumacher (2008) for technical details. Use of (11) as an end point in COVID‐19 treatment trials accounting for competing events has recently been considered by McCaw et al. (2020b). Study planning of some recent randomized clinical trials on treatment of COVID‐19 was also based on assumptions on median times to clinical improvement (Cao et al., 2020; Li et al., 2020). This will be discussed in Section 4.1.

### 3.4 Death after improvement or recovery: Illness–death model

For instance, McCaw et al. (2020b) broach the issue of longer follow‐up in future COVID‐19 treatment trials and its impact on meaningful outcomes including time to death. Here, one aspect is that prolonged survival on [0,τ], where, for example, τ is 28 days, does not benefit patients (Tan, 2020). The aim of the present subsection is to briefly outline how the competing events framework may be extended to also handle death events possibly after improvement or recovery during a longer follow‐up. To this end, define for finite times t the transition hazard

(12) $$\alpha_{12}\left(t;\vartheta \right)=\lim_{\Delta t↘0}\frac{P(X(t+\Delta t)=2\;|\;X(t-)=1,\vartheta <t,\vartheta )}{\Delta t},$$

where we now also model 1→2 transitions along the dashed arrow in Figure 1. The model has recently been used to jointly model time‐to‐progression (not a favorable outcome, of course) or progression‐free‐survival and overall survival by Meller et al. (2019). The model is time‐inhomogeneous Markov, if $\alpha_{12}\left(t;\vartheta \right)$ does not depend on the finite value of ϑ. Again a proportional hazard model may be fit to the transition hazard, possibly also modeling departures from the Markov assumption, but the interpretation of probabilities arguably is more accessible. One possible outcome could be the probability to be alive after recovery, that is, P(X(t)=1) over relevant time regions. In the context of clinical trials such outcomes have recently been advocated by Sommer et al. (2018) for treatment trials for severe infectious diseases and by Bluhmki et al. (2020) for patients after stem cell transplantation whose health statuses may switch between favorable and less favorable. Schmidt et al. (2020) have recently used such a multistate model in a retrospective cohort study on COVID‐19 patients, modeling oxygenation and intensive care statuses. For the statistical analysis, the authors used both Cox models of the transition hazards and reported estimated state occupation probabilities and “average” occupation times.

## 4 SOME DESIGN CONSIDERATIONS

Following on from the consideration of the choice of outcomes, their analysis, and interpretation in COVID‐19 trials, we now look into the consequences a particular choice of outcome has for the design of the trial. We start with sample size considerations and then comment on the use of adaptive designs, in particular sample size recalculation.

### 4.1 Power and sample size considerations

For a randomized controlled trial investigating the effect of a COVID‐19 treatment on clinical improvement or recovery of patients (addressing features (i) and (ii) of a successful treatment mentioned above) or death (addressing feature (iii) of a successful treatment), various approaches are conceivable. As described above, the time horizon τ considered is usually short, often 28 days. Therefore, it can reasonably be assumed that the recording of outcomes of interest such as hospitalization, ventilation, clinical symptoms, and death is complete. In this situation, an ordered categorical endpoint as the eight‐point ordinal scale proposed in the master protocol of the WHO (2020) and, for example, used in a seven‐point version in the trial by Goldman et al. (2020) at a prespecified time point (e.g., 28 days) or a simpler binary endpoint, as for example, death or clinical recovery as defined by a dichotomized version of the ordinal scale can be used as, for instance, in Lee et al. (2020). Endpoints captured at a prespecified time point would address above mentioned features (i) and (iii) of a successful treatment, but would not focus on feature (ii) of expediting recovery. An ordered categorical endpoint might be analyzed, under the proportional odds assumption, with a proportional odds model, for which sample size planning can be based on the formula proposed by Whitehead (1993). Under more general assumptions, the treatment groups might be compared with respect to an ordinal outcome by a nonparametric rank‐based approach using, for example, the Wilcoxon rank sum test and the so‐called probabilistic index or relative effect as effect measure (Kieser et al., 2013). The sample size can then be calculated using the formula provided by Noether (1987) or subsequent refinements using the variance under the alternative (Vollandt & Horn, 1997) or extensions to a variety of alternative hypotheses (Happ et al., 2019). A binary endpoint would commonly be analyzed using a logistic regression model and sample size planning can be based on formula (2) in Hsieh et al. (1998).

Even if the recording of the endpoints of interest can be assumed to be complete, it may be desirable to analyze not just the occurrence of the endpoint within the specified time period, but the time to the occurrence of the endpoint. This is mainly for three reasons. First, as described in Section 2, a time‐to‐event analysis captures not only a difference between treatments with respect to the proportion of patients for whom the event had occurred (addressing feature (i) of a successful treatment) but also a difference between treatments with respect to the time of occurrence (addressing feature (ii) of a successful treatment). This can be relevant even on a short time interval when the endpoint is, for example, time under mechanical ventilation, which has more severe adverse effects on patients health the longer it is required. Second, even if completeness of data over the time period τ is assumed, individual patients might be lost to follow‐up, which can be handled by a time‐to‐event analysis being able to include censored observations. Third, if interim analyses have to be conducted, which may often be the case in COVID‐19 trials, data of all patients can be included by censoring observations of patients with incomplete follow‐up appropriately in time‐to‐event analyses (Dodd et al., 2020).

In time‐to‐event analyses, we can model the effect of a treatment on the (event‐specific) hazard (3) or on the cumulative event probability (4) of experiencing the event of interest. In the presence of competing events, the cumulative event probability (4) depends on the event‐specific hazard for the event of interest and that one for the competing event as outlined in Section 3.1. As a consequence, the following conclusions can be drawn (Beyersmann et al., 2012). If treatment as compared to control leads to a decrease (or increase) in the cumulative probability of the event of interest, this could be for two reasons. It can be due to a direct (e.g., physiological) effect of treatment on the event‐specific hazard of the event of interest, or due to an effect on the event‐specific hazard of the competing event. Based on the analysis of the cumulative probability of the event of interest alone, it is difficult to capture the treatment mechanism resulting in a difference in event probabilities between treatment and control groups, since various treatment mechanisms can lead to the same difference in event probabilities. As a consequence, it is usually recommended to conduct three analyses for a complete understanding of treatment mechanisms, namely comparisons between treatment and control with respect to the event‐specific hazard of the event of interest, the event‐specific hazard of the competing event, and the cumulative event probability of the event of interest (Latouche et al., 2013).

In the planning of a clinical trial, one usually has to prespecify one treatment effect to be analyzed by one primary analysis (Baayen et al., 2019). In the following, we discuss the different approaches of focusing on the event‐specific hazard or on the cumulative event probability for the situation of our competing events model in Figure 1 where the event of interest is recovery from COVID‐19 and the competing event is death without prior recovery.

For a comparison of treatment groups with respect to the event‐specific hazards, the parameter of interest is the event‐specific hazard ratio


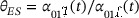

with 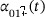 denoting the event‐specific hazard of the treatment group and 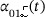 denoting the event‐specific hazard of the control group. Here, we are using Sütterlin script of the letters T and C to denote treatment and control to avoid confusion with event time *T* and censoring time *C*. For a comparison of treatment groups with respect to the cumulative probability of the event of interest, the parameter of interest is the subdistribution hazard ratio


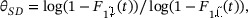

which follows from (6) under the assumption of proportional subdistribution hazard functions, with 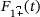 and 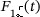, denoting the cumulative probability of the event of interest in the treatment group and control group, respectively. In the situation considered, where the event of interest is a favorable event such as recovery, for both quantities $\theta_{ES}$ and $\theta_{SD}$ superiority of treatment versus control is represented by a value larger than 1.

Whatever the planned analysis, that is, analysis of the event‐specific hazard ratio $\theta_{ES}$ or analysis of the subdistribution hazard ratio $\theta_{SD}$, sample size planning for a two‐sided level α test with power 1−β under an assumed hazard ratio θ is typically based on the Schoenfeld formula (Latouche et al., 2004; Ohneberg & Schumacher, 2014; Schoenfeld, 1981; Schoenfeld, 1983; Tai et al., 2018) for the total number of required recovery events

(13) $$E={\left(u_{1-\alpha /2}+u_{1-\beta }\right)}^{2}/\left[p\left(1-p\right){\left(\log\theta \right)}^{2}\right]$$

with p denoting the probability of being in treatment group T, and $u_{1-\gamma }$ denoting the (1−γ)‐quantile of the standard normal distribution. The total number of patients to be randomized can then be calculated as N=E/Ψ, where Ψ denotes the probability of observing a recovery event. In the absence of censoring, as assumed in our situation of a short planned trial duration of say 28 days, Ψ can be calculated as

(14)

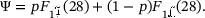

Although for the analysis of the event‐specific hazard ratio and the analysis of the subdistribution hazard ratio, the same formula for sample size calculation is often used, sample size planning, statistical analyses, and interpretation of results are different, as $\theta_{ES}$ and $\theta_{SD}$ represent different parameters as described above. Another issue is that Schoenfeld's formula assumes identical censoring distributions in the treatment groups; see Schoenfeld (1981). This assumption is well justified for time to an all‐encompassing endpoint and, technically, it lends itself to a particularly simple approximation of the covariation process of the log‐rank statistic underlying Schoenfeld's formula. It does, however, have further implications in the presence of competing events.

We will illustrate this for the simplistic assumption of constant event‐specific hazards of experiencing a recovery as the event of interest in treatment and control groups, 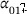 and 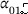, and of experiencing the competing event death without prior recovery in treatment and control groups, 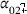 and 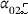. Hence, the event‐specific hazard ratios of recovery and of death without prior recovery are then given by 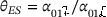 and 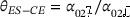, respectively. Under the constant hazards assumption, the cumulative probability of recovery in treatment group *k*, k=T,C, at time *t* is given by

(15) $$F_{1k}\left(t\right)=\frac{\alpha_{01k}}{\alpha_{01k}+\alpha_{02k}}\left[1-\exp\left(-\left(\alpha_{01k}+\alpha_{02k}\right)t\right)\right],$$

and the cumulative probability of death without prior recovery in treatment group k, k=T,C, at time *t* is given by

(16) $$F_{2k}\left(t\right)=\frac{\alpha_{02k}}{\alpha_{01k}+\alpha_{02k}}\left[1-\exp\left(-\left(\alpha_{01k}+\alpha_{02k}\right)t\right)\right].$$

The above formulae are special cases of (4). Under the assumption of constant event‐specific hazards, the limit of, for example, $F_{1k}\left(t\right)$ for t→∞ is

$$\frac{\alpha_{01k}}{\alpha_{01k}+\alpha_{02k}},$$

the relative magnitude of the event‐specific hazard of a type 1 event divided by the all‐events hazard. This quotient is multiplied with the usual formula of one minus the survival function under constant hazards.

Assuming Cox proportional hazards models for the event‐specific hazards, but not necessarily constant event‐specific hazards, the cumulative probability of recovery in treatment group T is given by

(17)

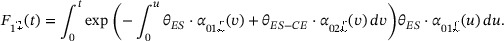

This equation slightly simplifies under constant hazards to


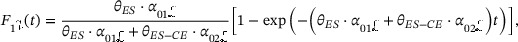

which is the same as (15).

Under the constant hazards assumption, Table 1 shows for different scenarios of assumed event‐specific hazards of recovery and death in treatment and control groups and associated event‐specific hazard ratios of recovery and death, the resulting cumulative event probabilities at time point 28 days and the resulting subdistribution hazard ratios at time point 28 days. Parameters were chosen to reflect similar scenarios as present in the recently published randomized clinical trials on COVID‐19 therapies, where observed probabilities of recovery were around 0.5–0.8 and observed probabilities of mortality were around 0.15–0.25 (Beigel et al., 2020; Cao et al., 2020; Li et al., 2020; Wang et al., 2020). Scenarios are shown under the constant hazards assumption with the main aim to illustrate how event probabilities and subdistribution hazard ratios result from two event‐specific hazard ratios and two baseline hazards. It can be seen that the same subdistribution hazard ratio can result from different combinations of event‐specific hazard ratios. Calculations for the more general case of proportional hazards could be derived from Equation (17) using numerical integration and would lead to similar insights.

**TABLE 1:** Event‐specific hazard ratios and the subdistribution hazard ratio at time 28 with respect to recovery for different scenarios under the constant hazard assumption

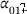	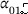	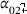	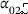	$\theta_{ES}$	$\theta_{ES-CE}$	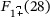	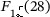	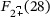	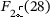	$\theta_{SD}\left(28\right)$
0.04	0.04	0.01	0.01	1.00	1.00	0.60	0.60	0.15	0.15	1.00
0.04	0.04	0.01	0.02	1.00	0.50	0.60	0.54	0.15	0.27	1.18
0.04	0.04	0.02	0.01	1.00	2.00	0.54	0.60	0.27	0.15	0.85
0.06	0.04	0.01	0.01	1.50	1.00	0.74	0.60	0.12	0.15	1.44
0.06	0.04	0.01	0.02	1.50	0.50	0.74	0.54	0.12	0.27	1.71
0.06	0.04	0.02	0.01	1.50	2.00	0.67	0.60	0.22	0.15	1.20
0.08	0.04	0.01	0.01	2.00	1.00	0.82	0.60	0.10	0.15	1.84
0.08	0.04	0.01	0.02	2.00	0.50	0.82	0.54	0.10	0.27	2.17
0.08	0.04	0.02	0.01	2.00	2.00	0.75	0.60	0.19	0.15	1.51
0.04	0.06	0.01	0.01	0.67	1.00	0.60	0.74	0.15	0.12	0.69
0.04	0.06	0.01	0.02	0.67	0.50	0.60	0.67	0.15	0.22	0.83
0.04	0.06	0.02	0.01	0.67	2.00	0.54	0.74	0.27	0.12	0.59
0.04	0.08	0.01	0.01	0.50	1.00	0.60	0.82	0.15	0.10	0.54
0.04	0.08	0.01	0.02	0.50	0.50	0.60	0.75	0.15	0.19	0.66
0.04	0.08	0.02	0.01	0.50	2.00	0.54	0.82	0.27	0.10	0.46

Note that the aim of the table is to illustrate possible constellations of the situation at hand, including some for which one would not plan a trial. To illustrate, when the event‐specific recovery hazards in treatment and control are identical $\left(\theta_{ES}=1\right)$, a decreasing effect of treatment as compared to control on the event‐specific death hazard $\left(\theta_{ES-CE}<1\right)$ leads to an increased cumulative recovery probability $\left(\theta_{SD}\left(28\right)>1\right)$, whereas an increasing effect of treatment as compared to control on the event‐specific death hazard $\left(\theta_{ES-CE}>1\right)$ leads to a decreased cumulative recovery probability $\theta_{SD}\left(28\right)<1$. Clearly, one would not plan a trial assuming the latter scenario, but it does illustrate that any competing events analysis is incomplete without a look at the competing event.

It is tempting to compare the magnitudes of $\theta_{ES}$ and $\theta_{ES-CE}$ with that of $\theta_{SD}\left(28\right)$. A situation of particular interest not just for this comparison arises when there is no treatment effect on the competing event‐specific hazard ratio, $\theta_{ES-CE}=1$. To begin, recall that any event‐specific hazards analysis is performed by handling observed competing events of the other type as censoring. Hence, assuming $\theta_{ES-CE}=1$ complies with the assumption of equal censoring mechanisms in the groups for using Schoenfeld's formula. Next, a proportional subdistribution hazards model will, in general, be misspecified assuming proportional event‐specific hazards as a consequence of (7). However, it has been repeatedly noted that ${\hat{\theta }}_{ES}\approx {\hat{\theta }}_{SD}$ if ${\hat{\theta }}_{ES-CE}\approx 1$ (Beyersmann et al., 2012; Saadati et al., 2018). This is mirrored in the table, in that scenarios with $\theta_{ES-CE}=1$ find comparable values of $\theta_{ES}$ and $\theta_{SD}\left(28\right)$. Note, however, that ${\hat{\theta }}_{SD}$ will estimate a time‐averaged subdistribution hazard ratio, averaged over the whole time span, computation of which requires numerical approximations (Beyersmann et al., 2012).

Equality (7) also illustrates that event‐specific and subdistribution hazards operate on different scales, and many authors have argued that the subdistribution hazard scale is more difficult to interpret; see Andersen & Keiding (2012) for an in‐depth discussion. We therefore refrain from further comparing the magnitudes of the different effect measures and rather continue with considering their impact on sample sizes following from Schoenfeld's formula.

For sample size planning of clinical trials where competing events exist, assumptions are usually based on the expected cumulative event probabilities (Baayen et al., 2019; Latouche et al., 2013; Schulgen et al., 2005; Tai et al., 2018). Under the constant event‐specific hazards assumption for both the recovery as well as the death without prior recovery hazard, the underlying hazards can be calculated from the cumulative event probabilities via Equations (15) and (16) as proposed by Pintilie (2002), Schulgen et al. (2005), Baayen et al. (2019) and Tai et al. (2018).

Table 2 contrasts for some scenarios of cumulative event probabilities similar to those of some recently published randomized clinical trials on COVID‐19 therapies the corresponding subdistribution recovery hazard ratio versus the event‐specific recovery hazard ratio calculated from the cumulative event probabilities under the constant event‐specific hazards assumption. Additionally, it is shown, which sample sizes would result if planning addresses the subdistributon recovery hazard ratio, the event‐specific recovery hazard ratio, or the odds ratio (of the binary endpoint recovery until day 28) for a randomized clinical trial which aims to show superiority of treatment as compared to control with respect to recovery from COVID‐19 with two‐sided type I error of 0.05 and power 0.8.

**TABLE 2:** Subdistribution hazard ratio and odds ratio (OR) at time 28, and event‐specific hazard ratio with respect to recovery derived from cumulative event probabilities under the constant event‐specific hazard assumption and resulting sample size when chosen as parameter for study planning with two‐sided type I error rate of 0.05 and power 0.8. Sample sizes $N_{ES}$ and $N_{SD}$ were calculated using Equations (13) and (14) and $N_{OR}$ was calculated using Equation (2) in Hsieh et al. (1998), all with p=0.5

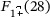	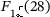	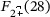	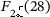	$\theta_{ES}$	$N_{ES}$	$\theta_{ES-CE}$	$\theta_{SD}\left(28\right)$	$N_{SD}$	OR (28)	$N_{OR}$
0.7	0.55	0.10	0.10	1.59	237	1.25	1.51	300	1.91	325
0.7	0.55	0.15	0.15	1.65	200	1.30	1.51	300	1.91	325
0.7	0.55	0.20	0.20	1.76	157	1.38	1.51	300	1.91	325
0.7	0.55	0.10	0.20	1.39	474	0.54	1.51	300	1.91	325
0.7	0.55	0.15	0.20	1.54	274	0.91	1.51	300	1.91	325

Table 2 deserves some discussion. To begin, we reiterate that Schoenfeld's formula assumes identical censoring mechanisms in the treatment groups. This is formally fulfilled when planning an analysis of $\theta_{ES}$ when $\theta_{ES-CE}=1$. In this case, a beneficial (harmful) effect on $\theta_{ES}$ directly translates into a beneficial (harmful) effect on the cumulative recovery probability. If the assumption of identical censoring mechanisms is violated, the reported sample sizes should serve as a starting point for simulation‐based sample size planning in practice. Ohneberg and Schumacher (2014) describe the use of simulation as a means for study planning with complex time‐to‐event outcomes including competing events. For the subdistribution approach, Latouche et al. (2004) find the use of Schoenfeld's formula to be quite reliable. This is of relevance for complete data on [0,28] with τ=28 as before and different probabilities of death $F_{2}\left(28\right)$ between groups. Here, the approach to handle deaths before time 28 as censoring at day 28 would imply identical (no) censoring on [0,28), but different censoring at time 28.

Next, analysis and sample size planning should not be guided by the required number of patients but by the interesting parameter. To this end, we reiterate that subdistributon times and, in particular, subdistributon hazards underly the analyses of recently COVID‐19 trials as outlined earlier, and Table 2 illustrates consequences of this choice. In Table 2, the entries 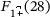 and OR(28) do not change, that is, are assumed to be the same across all scenarios, but the entries for $\theta_{ES}$ and $\theta_{ES-CE}$ do change, reflecting different entries 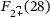 and $\theta_{ES-CE}$ can be modeled freely, that is, independent of each other, but, of course, the competing event probabilities do not share this property. In either case, the Table illustrates that careful planning requires assumptions on the event‐specific hazard or on the cumulative event probability of the competing event.

In some of the recently published randomized trials on the treatment of COVID‐19 (Cao et al., 2020; Li et al., 2020), sample size planning was performed in terms of assumed median times to clinical improvement. Both Cao et al. (2020) and Li et al. (2020) assumed for the control group a median time to clinical improvement of 20 days and a reduction of this time to 12 days in the active treatment group. For a two‐sided significance level of α=0.05 with a power of 80%, this resulted for the trial of Cao et al. (2020) to a total sample size of 160 patients under the assumption that 75% of the patients would reach clinical improvement and for the trial of Li et al. (2020) to a total sample size of 200 patients under the assumption that 60% of the patients would reach clinical improvement, both up to day 28. The proportions of patients with clinical improvement by day 28 were assumed to be different in both trials although identical median times to clinical improvement had been assumed. This could be due to different assumptions regarding the expected mortality rates not mentioned explicitly. From these specifications, we speculate that an exponential distribution for time to clinical improvement had been assumed leading to an event‐specific hazard ratio of 1.66 and a required number of patients experiencing the event clinical improvement of 120, which leads to the above‐mentioned patient numbers under the assumed proportions of clinical improvement by day 28. We note that in the statistical analysis of the trials, parameters were estimated from the subdistribution time, that is, the subdistribution hazard ratio and median times to clinical improvement based on quantity (11), being not quite consistent with the methods used for sample size calculation.

If the aim is to increase, say, the number of recoveries and to obtain these recoveries in a shorter time, the primary analysis may target the cumulative recovery probability as a function of time. Assuming that all or almost all patients experience one of the competing outcomes on [0,τ], it will suffice to target this probability because an increase of the recovery probability would then protect against a harmful effect on mortality. One possibility to demonstrate both an increase of the cumulative recovery probability and a shorter time to recovery is to establish a subdistribution hazard ratio larger than one. However, the interpretation of the subdistribution hazard is not straightforward, and an alternative would be a transformation model of the cumulative recovery probability using a logistic link (Eriksson et al., 2015) or a comparison of the cumulative recovery probabilities using confidence bands (Bluhmki et al., 2020).

When the cumulative recovery probability on [0,τ] is the target parameter, we see in Table 2 no large difference in the calculated sample size for the subdistribution hazard ratio based on (13) and (14) as compared to the calculated sample size for the odds ratio of the binary endpoint based on Formula (2) in Hsieh et al. (1998). When no competing events are present, it had been shown by Annesi et al. (1989) that the efficiency of an analysis with logistic regression is high as compared to an analysis with the Cox regression in the situation of a low event rate. We are not aware of a similar efficiency investigation comparing the Fine and Gray model with the logistic model in the presence of competing events. Formula (7) indicates that the subdistribution hazard is lower than the event‐specific hazard, so arguments related to low event rates could possibly translate.

Table 2 contrasts the sample size calculation with respect to the event‐specific hazard ratio, the subdistribution hazard ratio, and the odds ratio with respect to recovery. Another option would be choosing an ordinal categorical endpoint as, for example, the eight‐point ordinal scale proposed in the master protocol of the WHO (2020) or a version with fewer categories at a specified point in time (e.g., day 28) as target parameter for an analysis with the proportional odds model. In general, this would imply the assumption of a constant odds ratio for each one‐unit change in the ordinal scale. As an important consequence, it would not be consistent with an assumption of the superiority of treatment versus control with respect to the probability of recovery and the simultaneous assumption of identical probabilities of death as given in the first three rows of Table 2. For the situation given in Table 2 (increase of the recovery probability from 0.55 under control vs. 0.70 under treatment, odds ratio = 1.91), this would imply a decrease of the probability of the competing event death from 0.2 under control to 0.116 under treatment. Taking this in mind, the analysis of an ordinal endpoint would lead to a certain decrease in sample size as compared to the binary endpoint shown in Table 2, with the amount of decrease depending on the number of categories and the distribution of patients to the categories. As an example approximately comparable to the fourth row in Table 2, the planning for a trial with an ordinal endpoint with four categories with assumed proportions in control of 0.55, 0.12, 0.13, and 0.2 (with recovery as the highest category) would lead to a sample size of 300, whereas four categories with assumed proportions in control of 0.3, 0.25, 0.2, and 0.2 (with recovery as a combination of the highest and second highest category) would lead to a sample size of 245 (calculated using the formula by Whitehead, 1993). But as already outlined earlier, all these endpoints address different aspects of the treatment effect, which has to be taken into account. A summary of the different aspects of binary, ordinal and time‐to‐event endpoints in COVID‐19 trials is also given in Table S2 of Dodd et al. (2020) and in a similar situation in severe influenza in Peterson et al. (2019).

### 4.2 Sample size recalculation

As we have seen in Section 4.1, the sample size or power calculations rely on a number of assumptions. In particular, in an epidemic situation such as the ongoing COVID‐19 pandemic, there is no or very little prior knowledge regarding relevant parameters. In the context of COVID‐19 treatment trials considered here, these include treatment effects in terms of, for example, subdistribution hazard ratios or odds ratios but also potentially a range of nuisance parameters such as event probabilities regarding events of interest such as recovery, or competing events such as death. Sample size recalculation procedures were suggested to deal with this type of uncertainty and to make trials more robust to parameter misspecification in the planning phase (see, e.g., Mütze & Friede, 2021, for a recent overview). Generally, two broad classes of procedures are distinguished, namely sample size recalculation based on nuisance parameters and effect‐based sample size recalculation. Below, we comment on how these could be applied in the context of COVID‐19 treatment trials.

Designs with sample size recalculation based on nuisance parameters are also known as internal pilot study designs (Wittes & Brittain, 1990). The general procedure consists of the following steps: (i) a conventional sample size calculation is carried out at the design stage as outlined in Section 4.1; (ii) part way through the trial the nuisance parameters are estimated from the available data, and the sample size is recalculated based on these estimates; and (iii) in the final analysis, the combined sample of the internal pilot study and the remaining trial are analyzed. Nuisance parameters such as event probabilities might relate to the control group or the overall study population across the treatment groups. The latter can obviously be estimated from noncomparative data during the ongoing trial and does not require any unblinding. Therefore, it is often the preferred option, in particular in trials with regulatory relevance (EMA, 2007; FDA, 2018).

With a binary outcome such as recovery, the overall event probability could be considered the nuisance parameter, which can be estimated from the overall sample combined across the treatment arms. Gould (1992) provides sample size recalculation formulae based on the overall event probability for the odds ratio (considered above) as effect measure but also relative risks and risk differences. The way the treatment effect is specified is crucial here since it is kept fixed in the sample size recalculation. For instance, with the odds ratio as effect measure and event probabilities below (above) 0.5 a lower (higher) than expected event probability results in a sample size increase, whereas with a risk difference the sample size would be decreased.

Under the proportional odds model, the blinded procedure for binary outcomes can be extended to ordinal outcomes such as the scale suggested in the WHO master protocol (WHO, 2020). Rather than estimating the event probability from the sample pooled across the treatment arms, the distribution of the ordinal outcome across the outcome categories is assessed in the pooled sample (Bolland et al., 1998). Then the group‐specific distributions can be determined under the proportional odds model and the assumed odds ratio for the treatment effect. Hence, the sample size can be recalculated by plugging these estimates into the sample size formula by Whitehead (1993).

Guidance on blinded sample size recalculation procedures in time‐to‐event trials is provided in Friede et al. (2019) and references therein. In designs with flexible follow‐up times, the procedures would consider the recruitment, event, and censoring processes. In the situation considered here, trials are likely to use a fixed follow‐up design following all patients up to τ, say τ=28 days. Hence, the probabilities of the event of interest and the competing event would be estimated by the Aalen–Johansen estimator from the pooled sample in a blinded review. These findings would then be used to update the initial sample size calculation using the formulae provided in Section 4.1. The use of the Aalen–Johansen estimator is crucial here. Although follow‐up will eventually be complete in the final analysis, administrative censoring of some patients prior to τ is very likely at an interim time point. Therefore, the strategy of using the Kaplan–Meier estimator with observations of patients who died being censored not at the time of death but at τ would not be appropriate here, since the additional administrative censoring cannot be dealt with appropriately.

In the so‐called internal pilot study designs, the sample size calculation is typically carried out at a single time point during the study. Of course, the choice of the time point has implications for characteristics of the design such as the sample size distribution and power. Given the large uncertainty especially in an epidemic situation, the recommendation would be to consider repeated recalculations based on blinded data. Since the blinded procedure is fairly uncritical in terms of logistics and type I error rate inflation, this would be appropriate. Actually, the nuisance parameters could even be monitored in a blinded fashion from a certain point in time onwards, which should be rather early in situations of great uncertainty regarding the nuisance parameters. These procedures are also known as blinded continuous monitoring (Friede & Miller, 2012). In fact, this is typically done in event‐driven trials where the total number of events across both treatment arms is monitored. Since this principle can be transferred to other types of outcomes (Friede et al., 2019; Mütze et al., 2020), it would also be applicable to the type of COVID‐19 treatment trials considered here.

Group sequential designs belong to the class of designs with effect‐based sample size adaptation. They are used in many disease areas including oncology as well as cardiovascular and cardiometabolic research. For binary outcomes or ordinal outcomes under the proportional odds model, the procedures are well established (Jennison & Turnbull, 2000). In the context of COVID‐19 treatment trials, the presence of competing events would need to be accounted for. Here we refer to Logan and Zhang (2013) for group sequential procedures in the presence of competing events. Classical group sequential designs, however, must proceed in a prespecified manner and the size of the design stages must not be based on observed treatment effects unless prespecified weights for the design stages are used (Cui et al., 1999). The latter procedure is equivalent to the inverse normal combination function by Lehmacher and Wassmer (1999). Some issues in this type of design with time‐to‐event outcomes were raised (Bauer & Posch, 2004), but are not a concern in designs typical for COVID‐19 trials with a fixed follow‐up time as long as the patients are stratified by design stages in the analysis with patients belonging to the design stage they were recruited in although the observation of the outcome might extend into subsequent stages (Friede et al., 2011; Magirr et al., 2016). For a very recent review on adaptive designs for COVID‐19 intervention trials, see Stallard et al. (2020).

## 5 DISCUSSION

In the COVID‐19 pandemic, the fast development of safe and effective treatments is of paramount importance. Severe forms of COVID‐19 require hospitalization and in some cases intensive care. In these settings, recovery, mechanical ventilation, mortality, etc. are relevant outcomes. From a statistical viewpoint, different approaches to their analysis might be meaningful. Here we argued that a successful treatment of COVID‐19 patients (i) increases the probability of a recovery within a certain time interval, say 28 days; (ii) aims to expedite recovery within this time frame; and (iii) does not increase mortality over this time period. We recommend that this is reflected in the analysis approach. An implication is that COVID‐19 treatment trials are cast in a competing risks framework which, in general, requires an analysis of all event‐specific hazards and all cumulative event probabilities for a complete picture. However, as argued above, the cumulative improvement or recovery probability over the course of time [0,τ] is a natural primary endpoint, with additional control of competing mortality at τ, where τ denotes a limited number of days such as 28. Furthermore, we made recommendations regarding the design of COVID‐19 trials with such outcomes. Since there is no previous experience with COVID‐19, sample size calculations have to be informed by data from related diseases. This results in considerable uncertainty which can be mitigated by appropriate adaptive designs including blinded sample size reestimation.

Thus, a key conclusion for trials evaluating treatments of patients suffering from severe forms of COVID‐19 within a time frame of, for example, 28 days is that censoring deaths on day 28, but not on the day of death, results in a competing risks analysis provided that there is no additional censoring.

The presence of competing outcomes raises the question of the potential need to account for multiple testing. Recall that a successful trial would prove superiority for the favorable event and at least no effect or noninferiority for the competing event. Parameterizing this problem using event‐specific hazards, we note that the event‐specific analyses are asymptotically independent. With the wish to control the probability of erroneously rejecting at least one of the two null hypotheses, we find that we may consider the product of the single rejection probabilities. Consequently, the product is less than the individual levels.

Vaccines against COVID‐19 have become available, and it is of interest to consider how thoughts expressed in the present paper relate to vaccination trials. As examples, we consider Baden et al. (2021) and Voysey et al. (2021) who both use Kaplan–Meier estimation for the cumulative event probability of COVID‐19 events and express vaccine efficacy using hazards based on a Cox model (Baden et al., 2021) or Poisson regression (Voysey et al., 2021). Because vaccination is a measure of prevention in these papers and not used therapeutically, death is an extremely rare competing the event, and no practically relevant difference between Aalen–Johansen and Kaplan–Meier is to be expected because of competing mortality. While death was hardly a competing event in these papers, censoring was an issue requiring the use of survival methodology. Interestingly, Voysey et al. (2021) report an event‐driven design with censoring induced by the observation of 53 COVID‐19 events, which formally leads to dependent times‐to‐event and times‐to‐censoring processes and requires subtle martingale methods for hazards. So, compared to COVID‐19 treatment trials, the focus appears to shift from competing events to censoring, but it is worthwhile to note that the subtlety required for hazard‐based analyses found in the present paper is still required for vaccination trials, too. To illustrate, Baden et al. (2021) report a point estimate of vaccine efficacy of 94.1% which, however, has no immediate interpretation as a proportion. Rather, vaccine efficacy had been defined as one minus the hazard ratio. Using 1−0.941=0.059 and writing θ for the true hazard ratio,


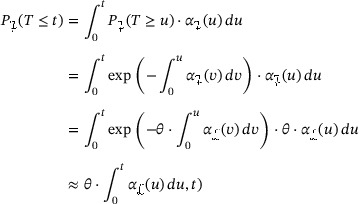

because, based on the empirical estimates in this trial, both θ and 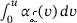 are very small. In fact, Baden et al. (2021) report a cumulative outcome probability in the placebo group that eventually reaches about 2.6% (see their Figure A). Hence,


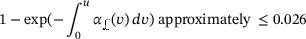

or, equivalently,


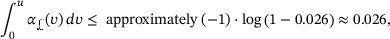

and we note that exp(−0.059·0.026)≈1. This also shows that the (estimated) cumulative hazard in the placebo group is so small that it approximately equals the cumulative outcome probability, and in summary,


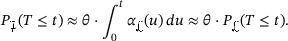

That is, with hindsight, we find that the reported vaccine efficacy may be interpreted via proportions, since both the estimated hazard ratio and the estimated outcome probabilities are very small.

The paper's focus is on 28‐day treatment trials with a time‐to‐event outcome and fixed follow‐up. Other design aspects that would be of interest include, but are not limited to, longer‐term effects and quality of life. In addition, we considered trials evaluating treatments of patients suffering from severe forms of COVID‐19. Of course, running trials in other disease areas have been affected by the pandemic. The issues and potential solutions are discussed in a recent paper by Kunz et al. (2020). Furthermore, we did not consider vaccine trials in more detail or diagnostic trials. Also, we assumed that event times were recorded on a continuous scale. In practice, however, this is strictly speaking not the case as event times might be reported in terms of days from randomization. In particular with shorter follow‐up times, this type of discreteness could be dealt with using appropriate models. For an overview, we defer the reader to Schmid and Berger (2020).

An illustration by applying the different approaches to analysis discussed here to a COVID‐19 trial would be rather desirable. At the time of writing, however, we did not have access to individual participant data from any COVID‐19 treatment trials that we could have used as an example here. We acknowledge that this is a limitation of our paper. Although data sharing is advocated, data availability remains an issue also with recent trials.

## CONFLICT OF INTEREST

The authors have declared no conflict of interest.

### OPEN RESEARCH BADGES

This article has earned an Open Data badge for making publicly available the digitally‐shareable data necessary to reproduce the reported results. The data is available in the Supporting Information section.

This article has earned an open data badge “**Reproducible Research**” for making publicly available the code necessary to reproduce the reported results. The results reported in this article could fully be reproduced.

## Supporting information

Supporting Information

## Data Availability

Data sharing does not apply to this article as no datasets were generated or analyzed during the current study.

## Appendix

The aim is to show that Equation (9), that is,

$$\hat{P}\left(T\leq t,X\left(T\right)=j\right)=1-\prod_{u\leq t}\left(1-\frac{\Delta N_{01}\left(u\right)}{\tilde{Y}\left(u\right)}\right),$$

holds. In words: The previous display states that the standard Aalen–Johansen estimator of the cumulative improvement (or recovery) probability accounting for the competing risk death equals one minus the Kaplan–Meier estimator based on the censored subdistribution times, assuming that the latter are censored solely at τ as a consequence of death before τ.

Checking the increments of any Kaplan–Meier‐type estimator, we find that the right‐hand side of the previous display equals

(A.1)
$$\sum_{u\leq t}\left\{\prod_{v<u}\left(1-\frac{\Delta N_{01}\left(v\right)}{\tilde{Y}\left(v\right)}\right)\right\}\frac{\Delta N_{01}\left(u\right)}{\tilde{Y}\left(u\right)}.$$

Now, $\tilde{Y}$ is a left‐continuous “at‐risk” process which includes all previous deaths. Introducing

$$N_{02}\left(t-\right)=\text{no.}\;\text{of}\;0\to 2\;\text{transitions}\;\text{on}\;\left(0,t\right),$$

we find that the product in the curly braces of Equation (A.1) has factors of the form

$$\frac{Y\left(v\right)+N_{02}\left(v-\right)-\Delta N_{01}\left(v\right)}{Y\left(v\right)+N_{02}\left(v-\right)},$$

where Y is the usual at‐risk process. Assuming no censoring on [0,τ), we have that for two neighboring type 1 event times $v_{1}<v_{2}$, that is, $\Delta N_{01}\left(v_{1}\right)\neq 0\neq \Delta N_{01}\left(v_{2}\right)$ and $N_{01}\left(v_{1}\right)=N_{01}\left(v\right)$ for all $v\in [v_{1},v_{2})$,

$$Y\left(v_{1}\right)+N_{02}\left(v_{1}-\right)-\Delta N_{01}\left(v_{1}\right)=Y\left(v_{2}\right)+N_{02}\left(v_{2}-\right).$$

As a consequence, canceling the appropriate terms leads to

$$\prod_{v<u}\left(1-\frac{\Delta N_{01}\left(v\right)}{\tilde{Y}\left(v\right)}\right)=\frac{\tilde{Y}\left(u\right)}{n}$$

and

$$1-\prod_{u\leq t}\left(1-\frac{\Delta N_{01}\left(u\right)}{\tilde{Y}\left(u\right)}\right)=\frac{N_{01}\left(t\right)}{n},$$

that is, the number of type 1 events on [0,t] divided by sample size. It is well known that the Aalen–Johansen estimator also equals $N_{01}\left(t\right)/n$ in the absence of censoring, which completes the argument.
