# Supplementary material for: Design aspects of COVID‐19 treatment trials: Improving probability and time of favorable events
Source: Biom J. 2021 Oct 22;64(3):440–60. doi: 10.1002/bimj.202000359 (PMC8653377; doi:10.1002/bimj.202000359)
Supplement: Supplementary file 1 — Supporting Information [file BIMJ-64-440-s001.zip › Beyersmann_etal_Design_COVID19_Table1.rtf]

alpha_01T	alpha_01C	alpha_02T	alpha_02C	theta_ES	theta_ES_CE	F_1T(28)	F_1C(28)	F_2T(28)	F_2C(28)	theta_SD(28)	
0.04	0.04	0.01	0.01	1.00	1.00	0.60	0.60	0.15	0.15	1.00	
0.04	0.04	0.01	0.02	1.00	0.50	0.60	0.54	0.15	0.27	1.18	
0.04	0.04	0.02	0.01	1.00	2.00	0.54	0.60	0.27	0.15	0.85	
0.06	0.04	0.01	0.01	1.50	1.00	0.74	0.60	0.12	0.15	1.44	
0.06	0.04	0.01	0.02	1.50	0.50	0.74	0.54	0.12	0.27	1.71	
0.06	0.04	0.02	0.01	1.50	2.00	0.67	0.60	0.22	0.15	1.20	
0.08	0.04	0.01	0.01	2.00	1.00	0.82	0.60	0.10	0.15	1.84	
0.08	0.04	0.01	0.02	2.00	0.50	0.82	0.54	0.10	0.27	2.17	
0.08	0.04	0.02	0.01	2.00	2.00	0.75	0.60	0.19	0.15	1.51	
0.04	0.06	0.01	0.01	0.67	1.00	0.60	0.74	0.15	0.12	0.69	
0.04	0.06	0.01	0.02	0.67	0.50	0.60	0.67	0.15	0.22	0.83	
0.04	0.06	0.02	0.01	0.67	2.00	0.54	0.74	0.27	0.12	0.59	
0.04	0.08	0.01	0.01	0.50	1.00	0.60	0.82	0.15	0.10	0.54	
0.04	0.08	0.01	0.02	0.50	0.50	0.60	0.75	0.15	0.19	0.66	
0.04	0.08	0.02	0.01	0.50	2.00	0.54	0.82	0.27	0.10	0.46	
