# Supplementary material for: Design aspects of COVID‐19 treatment trials: Improving probability and time of favorable events
Source: Biom J. 2021 Oct 22;64(3):440–60. doi: 10.1002/bimj.202000359 (PMC8653377; doi:10.1002/bimj.202000359)
Supplement: Supplementary file 1 — Supporting Information [file BIMJ-64-440-s001.zip › Beyersmann_etal_Design_COVID19_Table2.rtf]

F_1T(28)	F_1C(28)	F_2T(28)	F_2C(28)	theta_ES	N_ES	theta_ES_CE	theta_SD(28)	N_SD	OR(28)	N_OR	
0.7	0.55	0.10	0.10	1.59	237	1.25	1.51	300	1.91	325	
0.7	0.55	0.15	0.15	1.65	200	1.30	1.51	300	1.91	325	
0.7	0.55	0.20	0.20	1.76	157	1.38	1.51	300	1.91	325	
0.7	0.55	0.10	0.20	1.39	474	0.54	1.51	300	1.91	325	
0.7	0.55	0.15	0.20	1.54	274	0.91	1.51	300	1.91	325	
